# The Influence of the Manufacturing Technology on the Mechanical Properties of Woven Jute Fiber-Reinforced Epoxy Composites

**DOI:** 10.3390/polym17121649

**Published:** 2025-06-13

**Authors:** Radu Negru, Alexandru-Viorel Coșa, Adrian Ianto, Bogdan Tătar, Robert-Cătălin Sîrbu, Dan-Andrei Șerban

**Affiliations:** 1 Department of Mechanics and Strength of Materials, Politehnica University Timișoara, 300006 Timișoara, Romania; radu.negru@upt.ro; 2 Department of Materials and Manufacturing Engineering, Politehnica University Timișoara, 300006 Timișoara, Romania; alexandru.cosa@upt.ro (A.-V.C.); bogdan.tatar@student.upt.ro (B.T.); 3 Department of Mechanical Machines and Transportation, Politehnica University Timișoara, 300006 Timișoara, Romania; adrian.ianto@student.upt.ro; 4 Department of Mechatronics, Politehnica University Timișoara, 300006 Timișoara, Romania; catalin.sirbu@student.upt.ro

**Keywords:** woven fiber composites, jute fibers, wet layup, vacuum-assisted resin infusion, tensile tests, mechanical properties, multi-scale modeling

## Abstract

In this work, the mechanical properties of jute fiber-reinforced polymers were investigated, considering two manufacturing technologies—wet layups and vacuum-assisted resin infusion—with the aim of developing cost-effective composite materials based on natural fibers. In the manufacturing process, two different types of resins were used, specific to each technology. Specimens measuring 25 mm × 200 mm were cut from the resulting laminates at three orientations (0°, 45° and 90° with respect with the weft orientation), and they were subjected to tensile tests. The results showed that resin infusion yielded superior stiffness and strength values when compared to the wet layup. Multi-scale modeling techniques were applied in order to estimate the properties of the fibers and evaluate the orthotropic properties of the composites, and virtual material models that included orthotropic elasticity and the anisotropic Hill plasticity formulation were developed and evaluated, managing to reproduce the experimental data using finite element analyses with decent accuracy.

## 1. Introduction

In recent years, the usage of woven fiber-reinforced polymers in structural applications (such as airplane fuselages, ship hulls, vehicle chassis, etc. [1,2]) has experienced a significant increase to the detriment of unidirectional fiber or chopped strand mat counterparts, due to their good mechanical properties [3,4], relatively easy ply stack design (woven fiber laminates usually consist of [0°/45°]_n_ stacks due to their orthogonal symmetry, when compared to the relatively wide array of orientation angles and symmetry conditions for long fiber composites [5,6]) and better drapability and stability during manufacturing [7,8] when compared to other composite reinforcements. Additional advantages include high availability and variety (including a wide array of weave types and densities [9,10,11]), with the main drawback being their increased purchase cost when compared to long fibers and chopped strand mats.

There are several manufacturing technologies associated with the fabrication of woven fiber composite parts, the main being wet layups, resin infusion, injection molding and pre-impregnate forming. The latter two are more restrictive, as injection molding requires expensive equipment and tools and is only suitable for thermoplastic polymer matrices while pre-impregnate sheets require an additional manufacturing process, special resins (that cure at high temperatures) and complex equipment (autoclaves) [2,4,12]. However, in some cases, their advantages overshadow the drawbacks, as injection molding can yield very high productivities (compared to other woven fiber composite manufacturing technologies) while pre-impregnates yield the best material properties (lowest matrix weight fraction) and shapability (extremely high stability during positioning in the mold and the ability to be cut into any required shape without fraying) [13,14,15].

Resin infusion is considered one of the optimal manufacturing technologies for composite materials, as it achieves a balance between the cost of production, the resulting material properties and surface finish. The infusion process can be performed using either pressure or vacuum [16,17,18]. Wet layups on the other hand are considered to be the most “primitive” and the cheapest manufacturing process for composites, but they generally yield the lowest mechanical properties and the worst surface finishes. However, these negative aspects can be improved through several additional steps that aim to reduce the resin content [19,20,21].

The main types of woven fibers used for composite materials are glass, carbon and aramid. Though they exhibit remarkable mechanical properties, there are growing concerns related to their impact on the environment. Regarding the sustainability of their manufacturing, the relatively high degrees of pollution, their non-renewable nature and energy consumption during processing are major concerns, with various measures being proposed to mitigate these effects, such as bio-based precursors or novel processing technologies [22,23,24,25]. Another issue is related to their end-of-life management and circularity, where limited options are available [26], the main countermeasures being lifetime extension and increases in durability and stability [26,27,28].

A step in countering this aspect is the use of natural fibers as reinforcements. Though their mechanical properties are considerably lower when compared to the most commonly used synthetic fibers (Table 1), their renewable aspects and possible compostability (if organic matrices are used) support their use as suitable alternatives [29].

The main goal of this study is to develop cost-effective composites based on woven natural fibers. In consequence, woven jute fibers were used as reinforcements due to their relatively low price (10% of the price of hemp cloth, 35% of the price of flax cloth and 65% of the price of cotton cloth) and decent mechanical properties (average values of 30 GPa stiffness and 580 MPa tensile strength [30,31,32,33]). Epoxy resins were used as the matrix for the two manufacturing technologies that were considered due to the relatively low costs associated with the equipment and consumables: wet layups and vacuum-assisted resin infusion. Investigation regarding the thickness, density and mechanical properties of the resulting materials was performed in order to determine the technology that produces optimal parts considering specific applications. Multi-scale modeling of the resulting materials was considered in order to evaluate the mechanical properties of the constituents (i.e., of the jute fibers) and the composites (i.e., shear moduli and Poisson ratios) that were not determined experimentally with the final goal of developing accurate virtual material models that can be employed in the finite element analyses of complex parts in order to evaluate their structural integrity during the design stage.

The number of previous studies performed on woven jute fiber-reinforced epoxy resins is reduced, with the literature survey only identifying tests on composites manufactured through wet layups, which yielded tensile moduli of 198–10,100 MPa (the lower bounds, determined by one study, are doubtful) and tensile strengths of 12.79–19.5 MPa [30,34,35].

## 2. Materials and Methods

**Figure 1:**
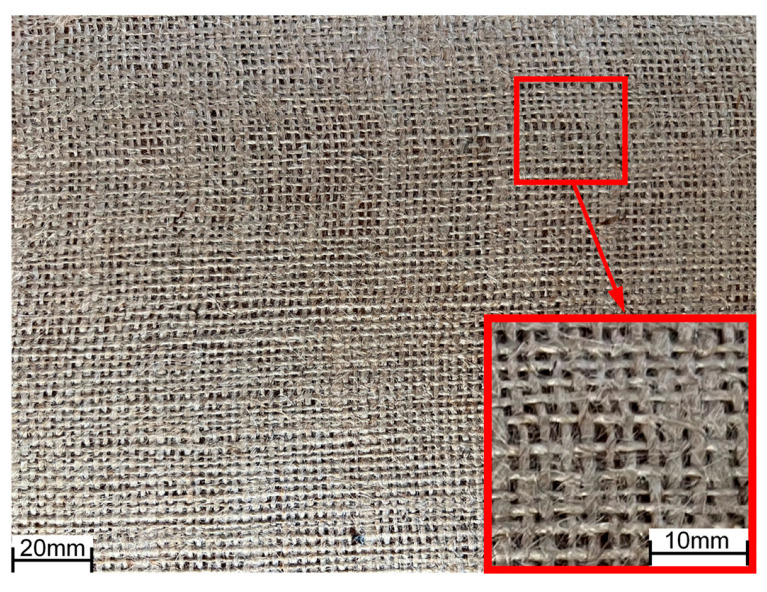
Jute fiber cloth.

Considering the recommendations of the European Commission regarding decarbonization policies [36], the reinforcement material, which consisted of a woven jute fiber in a plain pattern, was manufactured locally and supplied in a 1 m wide roll by Unico S SRL, Hunedoara, Romania. The fabric was characterized by a weft of 4.6 yarns·cm^−1^ and a weave of 4.8 yarns·cm^−1^, with a density of 350 g·m^−2^ [37]. The main destination of all the locally sourced jute fiber cloth is the manufacturing of textile sacks for the transportation of various agricultural goods. In consequence, the quality of the cloth is quite low, exhibiting an uneven distribution and significant variation in the thickness of weaves and wefts (as seen in Figure 1), which induces a certain degree of non-homogeneity, potentially causing a more pronounced scatter in results. The cloth was cut into 500 mm × 300 mm samples for the layup process.

In the manufacturing of the composites, two types of epoxy resins were used, specific to each manufacturing technology. For the wet layups, a bi-component (resin and hardener) with the commercial name Vosschemie^®^ Epoxy BK was used (provided by the BestTools SRL, Brașov, Romania), with the compound having a density of 1.075 g·cm^−3^ and viscosity ranging between 660 and 1100 mPa·s [38]. For the resin infusion process, a bi-component (resin and hardener) with the commercial name Easycomposites^®^ IN2 Epoxy Infusion Resin was used (provided by Easy Composites Ltd., Stoke on Trent, UK), with a density of 1.15 g·cm^−3^ and viscosity ranging between 200 and 450 mPa·s [39].

For both processes, a 15 mm thick glass mold was used (500 mm × 800 mm), on which a physical demolding agent (commercial name Globalwax 200 L) was applied in 3 stages.

**Figure 2:**
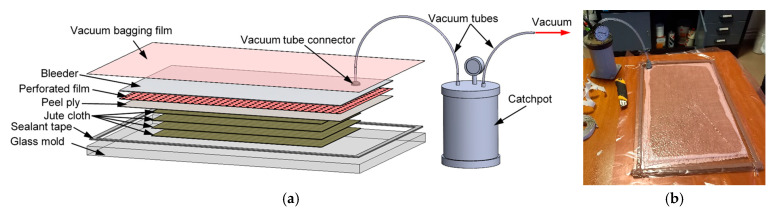
Wet layup manufacturing: schematic (**a**) and experimental setup (**b**).

The wet layup manufacturing process consisted in the successive application of the resin (using a brush) and the positioning of the cloth, for a total of four plies. After the application of the resin, a peel ply (to facilitate top demolding), a perforated release film (to limit the amount of resin pulled by the vacuum) and a bleeder cloth (to absorb the excess resin) were positioned on top of the layup. Finally, a nylon vacuum bagging film was attached to the mold using sealant tape and the full vacuum was pulled using a vacuum pump, silicone vacuum tubes and a silicone connector through a catchpot (used to capture any resin that might be pulled during the process, in order not to damage the pump; Figure 2a). After the full vacuum was reached, the vacuum tube was sealed using a metal clamp and the composite layup was left to cure under vacuum for 24 h at ambient temperature (20–23 °C) (Figure 2b).

**Figure 3:**
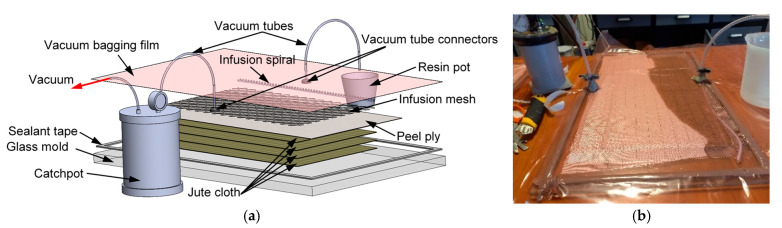
Vacuum-assisted resin infusion manufacturing: schematic (**a**) and experimental setup (**b**).

For the vacuum-assisted resin infusion process, for which the setup is presented in Figure 3a, the four woven jute plies were positioned on the mold and secured with adhesive tape and a peel ply was positioned on top. For an optimized resin distribution, a resin infusion mesh was positioned on top of the peel ply. Two silicone connectors were installed, one for the resin feed (that had an infusion spiral attached) and one for the vacuum. The mold was then sealed in a similar manner to the wet layup setup, the resin feed tube was sealed and the full vacuum was pulled in order to check for eventual leaks. Afterwards, the feed tube was cleared and the resin was infused (Figure 3b), with both tubes being sealed when the resin was distributed throughout the layup, and the plate was left to cure under vacuum for 24 h (20–23 °C).

**Figure 4:**
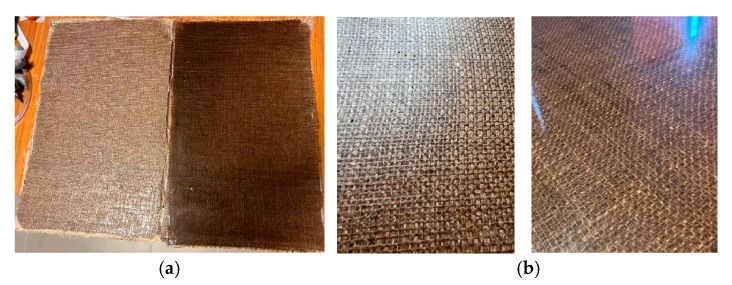
Visual comparison between the two resulting composite layups: overview (**a**) and detail (**b**).

A visual comparison between the resulting plates manufactured using the two different technologies is presented in Figure 4. It can be observed that the plate manufactured through infusion exhibits superior surface properties, with higher gloss and a significantly lower content of trapped air. The color difference is caused mostly by the resin shade.

From the plates, 25 mm × 200 mm specimens were cut using a rotary saw at 0°, 45° and 90° with respect to the weft orientation (five specimens for each configuration).

Each specimen was measured and weighed (three measurements for each characteristic) in order to determine the average density of each material (15 specimens for each material), with the results being presented in Table 2.

**Figure 5:**
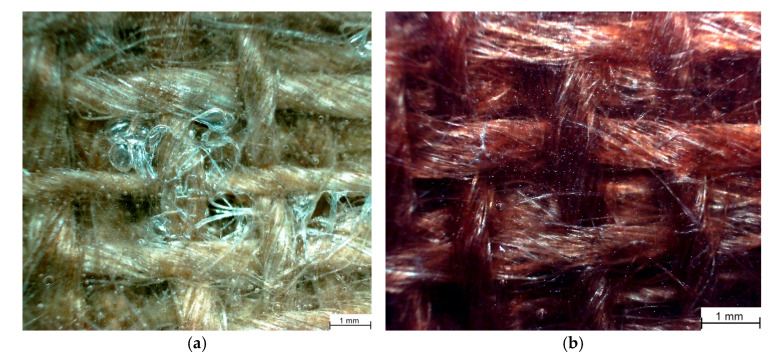
Microscopic analysis of the wet layup exhibiting local defects (**a**) and infusion (**b**) specimens.

It can be observed that the wet layup specimens exhibit higher thickness and lower density. This can be explained, on the one hand, by the larger density of the infusion resin and, on the other hand, by the higher air content in the wet layup specimens, as seen in Figure 5.

**Figure 6:**
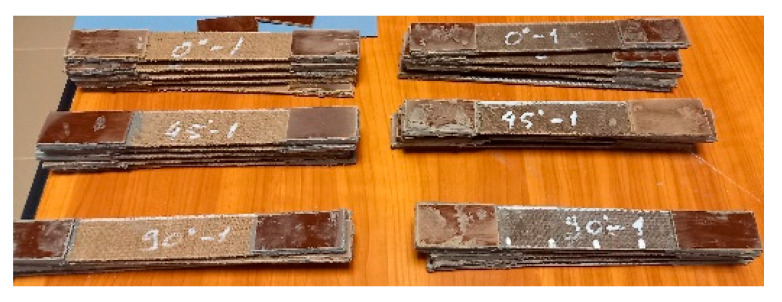
Test specimens with bonded textolite tabs, grouped by material and orientation.

After the measurements, at the ends of each specimen, textolite tabs were bonded using an epoxy-based adhesive, in order to avoid the stress concentration caused by the machine grip pressure during testing (Figure 6).

The experimental procedures consisted in tensile tests performed on a servo-hydraulic 150 kN Walter+Bai LFV-L testing machine (Walter+Bai AG, Löhningen, Schaffhausen, Switzerland) at ambient temperature, using a crosshead travel speed of 5 mm/min, with the strains being recorded using an incremental extensometer.

## 3. Results and Discussions

**Figure 7:**
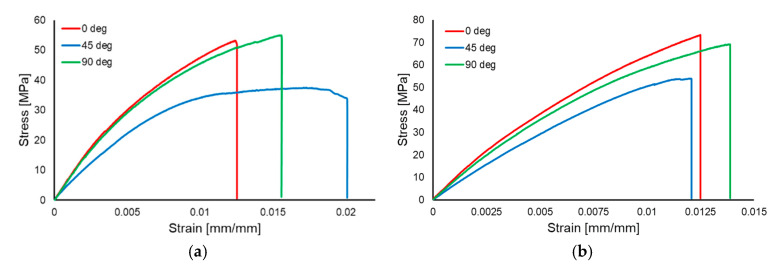
Stress–strain curves for the wet layup specimens (**a**) and for the infusion specimens (**b**).

Representative stress–strain curves for each orientation are presented in Figure 7a for the wet layup specimens and in Figure 7b for the infusion specimens.

**Figure 8:**
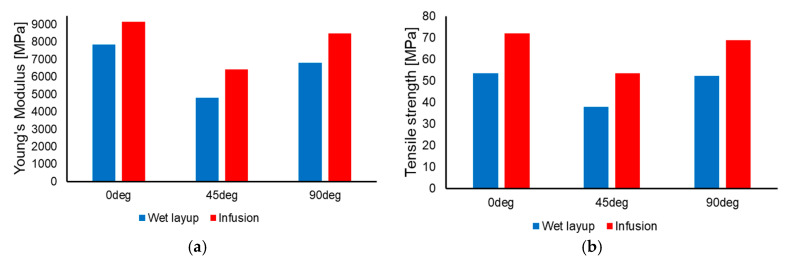
Comparison between the wet layup and infusion specimens for Young’s modulus (**a**) and tensile strength (**b**).

The average results for Young’s modulus and tensile strength are presented in Table 3 and in a graphic variation in Figure 8.

From the experimental results it can be observed that the woven jute fiber-reinforced epoxy exhibits characteristics similar to other woven fiber-reinforced polymers, with lower mechanical properties for the 45° specimens when compared to the 0° and 90° specimens. A certain degree of orthogonal anisotropy can be observed in both cases, due to the different spacing and the dimensional inconsistency of the yarns for the wefts and weaves.

**Figure 9:**
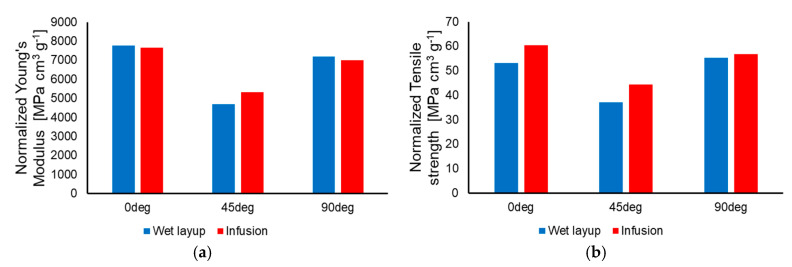
Comparison between the wet layup and infusion specimens for the normalized Young’s modulus (**a**) and normalized tensile strength (**b**).

The specimens manufactured through vacuum-assisted resin infusion exhibited superior properties when compared to the specimens manufactured with the wet layup technique. However, Table 1 shows that, on average, the infusion specimens present a higher density. In consequence, it is worthwhile to examine the mechanical properties of the materials normalized to their density (i.e., the stiffness and strength of specimens having the same length and weight). The normalized values are presented in Table 4 and a graphical variation in Figure 9, showing that the average values are closer and, in some cases, the wet layup specimens yield superior properties.

## 4. Multi-Scale Modeling

The multi-scale modeling presented in this section includes micromechanical modeling, property estimation through homogenization and macromechanical modeling, with the aims being twofold: the first is to estimate the stiffness of the jute fibers (since the literature values range from 10 GPa to 55 GPa [30,31,32,33]) and the second is to evaluate the shear modulus of the composites, since no shear tests were performed.

The first step required for micromechanical modeling is the determination of the fraction of fibers, resin and air in the composites. In terms of the volumes of the constituents, the relations can be expressed as

(1) $$\phi_{resin}+\phi_{fibers}+\phi_{air}=1$$

where $\phi_{resin}$, $\phi_{fibers}$ and $\phi_{air}$ are the volume fractions of the resin, fibers and air, respectively.

The main challenge for this class of materials is the estimation of the fraction of resin and air in each type of material. The jute density is reported to be around a value of 1.45 g·cm^−3^ [30,31,32,33]. Microstructural investigations (Figure 5) determined an average width of the yarn of 0.92 mm for the wet layup specimens and around 0.96 mm for the infusion specimens. Considering the average heights of the specimens, the number of plies and a uniform yarn crimp, the average heights of the yarns ${\hat{h}}_{yarn}$ were evaluated (Equation (2)), resulting in values of 0.287 for the wet layup and 0.268 for the infusion specimens.

(2) $${\hat{h}}_{yarn}=\frac{\hat{h}}{2\cdot n}$$

where $\hat{h}$ is the average height of the specimens for each type of material and n=4 is the number of plies in the specimens.

**Figure 10:**
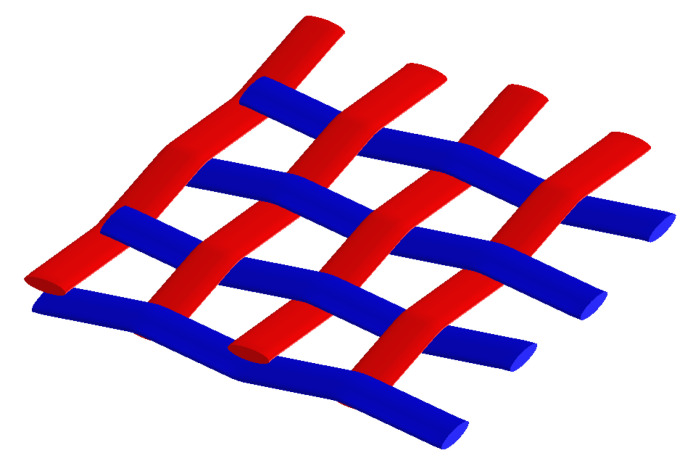
Representative volume element for the virtual jute fabric model.

In addition, in order to generate a microstructural unit-cell, the linear density of the yarn was evaluated, resulting in a value of 375 g·km^−1^. Based on this data, a representative volume element was generated using the commercial software MSC Software^®^ Digimat 2017.0, considering the fabric characteristics (plane weave, weave of 4.8 yarns·cm^−1^ and weft of 4.6 yarns·cm^−1^), depicted in Figure 10.

The software evaluated the jute fiber contents of the dry fabric (considering the fiber volume fraction in the yarn and the weave geometry), resulting in a fiber volume fraction $\phi_{fibers}$ of 0.295 for the wet layup and 0.338 for the resin infusion specimens. The higher value of the infusion specimens is most likely caused by the better compaction of the yarns, with the evacuation of the air in between the fibers during the manufacturing process.

In order to evaluate the mass fraction of the fibers $\mu_{fibers}$, the area density of the fabric $\rho^{area}$ and the average surface area of the specimens $\hat{A}\;$ (Table 1) were considered (Equation (3)), resulting in values of 0.532 for the wet layup samples and 0.539 for the infusion samples.

(3) $$\mu_{fibers}=\frac{4\cdot \rho^{area}\cdot \hat{A}}{{\hat{m}}_{specimen}}$$

where ${\hat{m}}_{specimen}$ is the average mass of the specimens for each material type.

Based on the mass fractions of the fibers $\mu_{f}$ and the densities of the resins $\rho_{resin}$, the volume fraction of the resins $\phi_{resin}$ was evaluated (Equation (4)), resulting in values of 0.576 for the wet layup and 0.658 for the infusion.

(4) $$\phi_{resin}=\frac{\rho_{resin}\cdot {\hat{m}}_{specimen}\left(1-\mu_{fibers}\right)}{{\hat{V}}_{specimen}}$$

where ${\hat{V}}_{specimen}=\hat{A}\cdot \hat{h}$ is the average volume of the specimens for each material type.

Considering the fiber and resin volume fraction values, the air volume fraction values were determined (Equation (1)), resulting in values of 0.129 for the wet layup and 0.041 for the resin infusion specimens. The evaluated air volume fractions are in agreement with the microscopic investigations regarding the structures of the composites (Figure 5).

**Figure 11:**
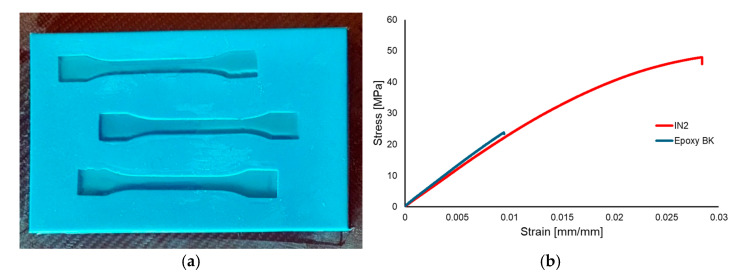
Resin ISO 527 specimen manufacturing (**a**) and representative stress–strain curves for the two resins (**b**).

In order to perform micromechanical analyses, the stiffness of the resins and that of the jute cloth need to be determined. For the resins, the mechanical properties were determined through tensile testing on ISO 527 [40] specimens (five specimens for each resin) on a Zwick/Roell Z005 machine (Zwick Roell, Ulm, Germany), which were obtained through the casting of the resins in silicone molds (Figure 11a), with the resulting stress–strain curves presented in Figure 11b and the average results and standard deviations presented in Table 5.

Since the literature values for the jute fibers’ Young’s moduli vary in such a large interval, the starting value was estimated to be an approximate value of 21.3 GPa using the rules of mixture:

(5) $$E_{fibers}^{max}=\frac{E_{composie}-\phi_{resin}E_{resin}}{\phi_{fibers}}$$

In consequence, the Young’s modulus of the fibers varied between 21 GPa and 18 GPa for both types of materials. Based on these values, the dimensions of the yarns (average measured width and evaluated height) and the yarn linear density, the software evaluated the mechanical properties of the yarns used in the simulations (axial, in-plane and transverse Young’s moduli and Poisson ratios), considering an elliptic cross-section.

The resins were modeled as isotropic with the effective Young’s moduli ${\tilde{E}}_{resin}$ estimated through homogenization from the values presented in Table 3 and the air volume content.

(6) $${\tilde{E}}_{resin}=\frac{\phi_{resin}E_{resin}}{\phi_{resin}+\phi_{air}}$$

**Figure 12:**
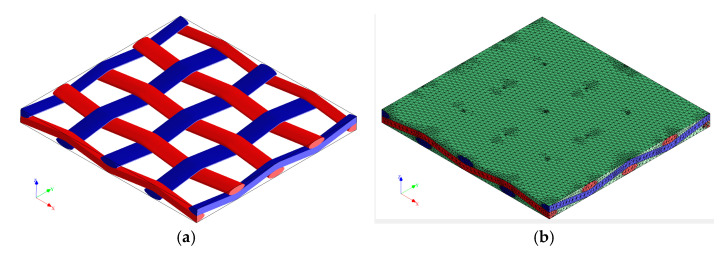
RVE geometry (**a**) and meshed model (**b**) for the finite element analysis.

The representative volume element (RVE) based on the theoretical geometry (Figure 10) used in the analyses is presented in Figure 12a, with the mesh, consisting of around 211,000 tetrahedral elements, presented in Figure 12b.

The models were subjected to periodic boundary conditions and loadings along all the major directions in order to determine the stiffness ($E_{1}$, $E_{2}$, $G_{12}$, $G_{23}$ and $G_{31}$) and Poisson ratios ($\nu_{12}$, $\nu_{13}$ and $\nu_{23}$) of the two types of composites.

**Figure 13:**
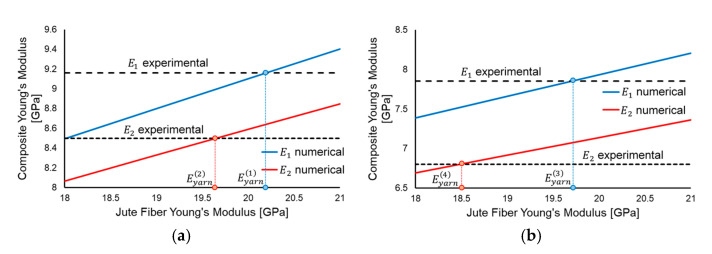
Young’s modulus variation comparison between the RVE analysis results and the experimental results for the wet layup (**a**) and resin infusion (**b**).

The results of the simulations determined a linear variation in the composites’ stiffness with the Young’s modulus of the fibers for both principal directions ($E_{1}$ and $E_{2}$), as depicted in Figure 13. The values for the jute fibers’ Young’s moduli ($E_{yarn}^{\left(i\right)}$) were determined through the intersection of the numerical variations (blue lines for $E_{1}$ and red lines for $E_{2}$) with the horizontal lines defined by the experimental values, yielding four distinct values: $E_{yarn}^{\left(1\right)}=20.2\;GPa$, $E_{yarn}^{\left(2\right)}=19.6\;GPa$, $E_{yarn}^{\left(3\right)}=19.7\;GPa$ and $E_{yarn}^{\left(4\right)}=18.5\;GPa$. These four different values are caused by two factors: (i) the simulations did not manage to capture the relatively high degree of anisotropy along the major axes that was determined experimentally, yielding one value that accurately predicts the Young’s modulus of the composite for the 0° orientation ($E_{yarn}^{\left(1\right)}$ and $E_{yarn}^{\left(3\right)}$) and another value for the 90° ($E_{yarn}^{\left(2\right)}$ and $E_{yarn}^{\left(4\right)}$) and (ii) the resin infusion simulations required lower values of fiber stiffness in order to obtain a match when compared to the wet layup simulations. In consequence, the numerically determined jute fiber Young’s modulus was considered to vary between the lowest and highest matching values, namely, 18.5 GPa and 20.2 GPa.

Considering the two distinct values for the fiber moduli that yielded matching composite moduli for each type of material, the shear properties and Poisson ratios were also set to vary in an interval, similarly to the jute fiber Young’s modulus. The lower bound was set to be the simulation result for the lower matching fiber moduli (i.e., simulations that used $E_{yarn}^{\left(2\right)}$ and $E_{yarn}^{\left(4\right)}$, respectively), while the upper bounds were imposed as the results for the highest matching fiber moduli (i.e., $E_{yarn}^{\left(1\right)}$ and $E_{yarn}^{\left(3\right)}$, respectively). These variations are presented in Table 6 for both materials.

The macromechanical analyses were performed in Abaqus 3Dexperience R2019 on flat shell parts of 25 mm×50 mm (representing an approximation of the surface enclosed by the extensometer). The elastic properties of the two material models consisted of orthotropic plane stress elasticity (Table 7) with the constants chosen based on the experimental data (average values for the Young’s moduli $E_{1}$ and $E_{2}$ from Table 3) and the numerical analyses (average values for the shear moduli and Poisson ratios from the intervals presented in Table 5).

**Figure 14:**
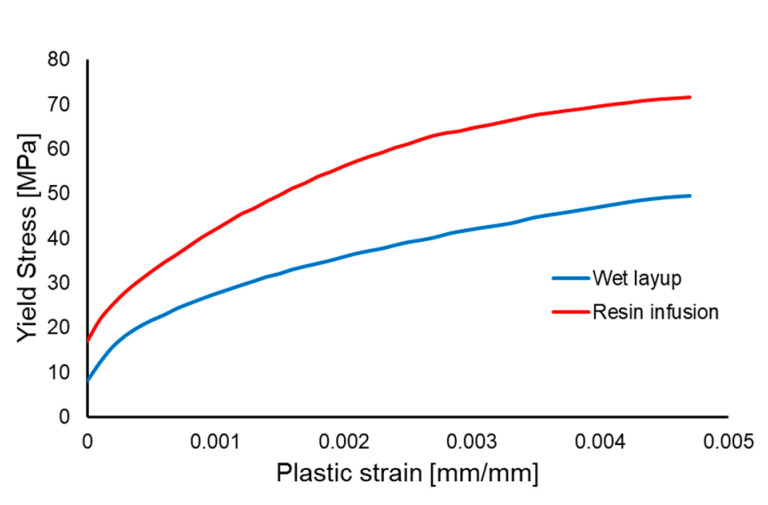
Yield stress–plastic strain curves for the two materials.

Given the non-linear characteristics of the experimental stress–strain curves, a multi-linear hardening plasticity model was included, using isotropic hardening and the Hill anisotropic formulation [6,41]. The hardening curves for the two materials (Figure 14) were determined from the true stress–logarithmic strain data derived from the experimental data of the 0° orientation tests [42].

The Hill plasticity definition requires the input of six coefficients, defined as

(7) $$\begin{array}{c} R_{11}=\frac{\sigma_{11}}{\sigma^{0}};\;R_{22}=\frac{\sigma_{22}}{\sigma^{0}};\;R_{33}=\frac{\sigma_{33}}{\sigma^{0}};\\ R_{12}=\frac{\tau_{12}}{\sigma^{0}};\;R_{13}=\frac{\tau_{13}}{\sigma^{0}};\;R_{23}=\frac{\tau_{23}}{\sigma^{0}}; \end{array}$$

where $\sigma^{0}$ is the yield stress of the hardening curve, $\sigma_{ii}$ are the yield stresses for the tensile tests at the given orientations and $\tau_{ij}$ are the yield stresses for the shear tests along the given directions. Since no shear tests were performed, the shear yield stress $\tau_{12}$ and shear yield strain $\gamma_{12}$ were evaluated from the tests performed at 45°, according to [43].

(8) $$\tau_{12}=\frac{\sigma_{x}}{2};\;\gamma_{12}=\varepsilon_{x}\left(1+\nu_{12}\right)$$

The resulting values for the R coefficients of the two materials are presented in Table 8.

**Figure 15:**
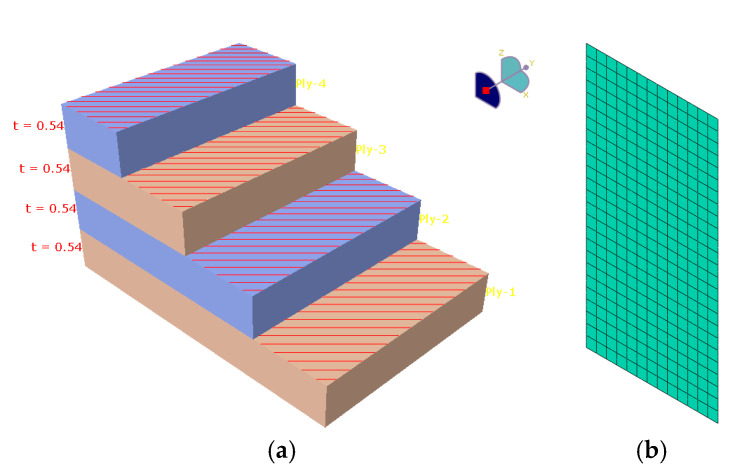
Example of a ply stack layup assigned as the material section for the shell elements (**a**) and the meshed geometry used in the numerical analyses (**b**).

A four-layer composite layup was applied to the shell (Figure 15a), while the mesh consisted of 325 s order quadratic shell elements (SR8) of 2 mm average length (Figure 15b). A *Y*-axis symmetry was imposed on the lower surface of the specimens, while a displacement was imposed on the top surface. The reactions in the nodes were used to evaluate the nominal stress in the specimens, while the displacement of the top surface was used in evaluating the nominal strains.

**Figure 16:**
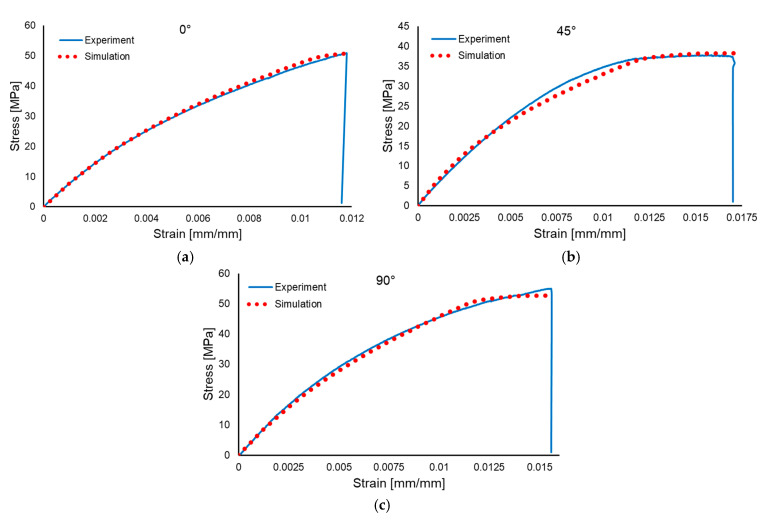
Comparison between experimental and numerical results for the wet layup: 0° orientation (**a**), 45° orientation (**b**) and 90° orientation (**c**).

**Figure 17:**
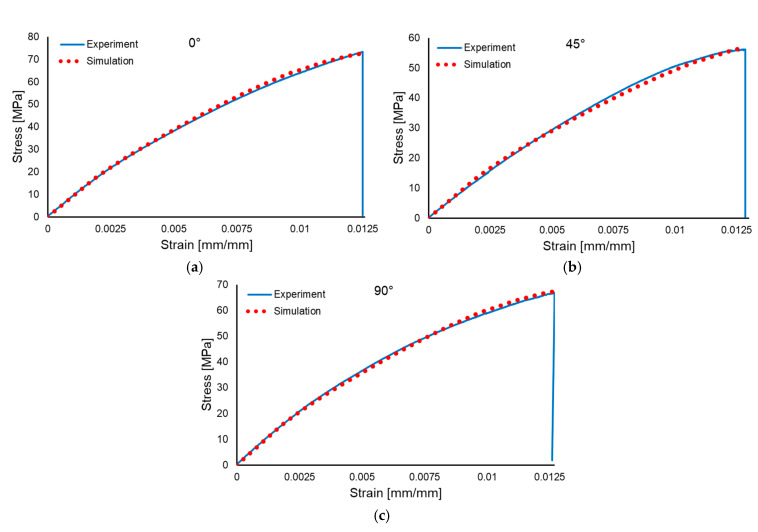
Comparison between experimental and numerical results for the resin infusion: 0° orientation (**a**), 45° orientation (**b**) and 90° orientation (**c**).

Three simulations were performed for each material type, using the orientations of the plies along 0°, 45° and 90°, respectively. The comparisons between the experimental and numerical stress–strain results are presented in Figure 16 for the wet layup material and in Figure 17 for the resin infusion material.

## 5. Conclusions

The experimental results from this study improve the limited knowledge regarding the use of woven jute fibers in the manufacturing of composite materials, with the two manufacturing technologies investigated in this work yielding decent results in terms of stiffness and strength when compared to other natural fibers. In consequence, considering its reduced cost, this type of reinforcement can be considered a suitable candidate for composite manufacturing, especially when biological fibers are involved. The main disadvantages related to the use of woven jute fibers are linked to the quality of the cloth. Even though the mechanical properties of the fibers are comparable to other natural fibers, the quality of the commercially available jute cloth is a concern, yielding lower values for the mechanical properties when compared to flax or hemp cloths and, in addition, a relatively large dispersion in results. Further improvements in the mechanical properties of such composites would imply the manufacturing of higher-grade cloths, with tighter and more uniform weaves (higher surface density, which would yield larger fiber content).

The vacuum-assisted resin infusion process yields superior mechanical properties to the wet layup process (14–19% larger Young’s modulus values and 24–29% larger tensile strength values), even though the infusion resin exhibits 6.71% lower stiffness when compared to the layup resin as well as a superior surface finish (Figure 4b), with a significantly lower amount of trapped air (4.1% air volume compared to 12.9%). However, if the overall mass of the component is prioritized (i.e., the ply stack of a component is designed with mass constraints instead of dimensional constraints), the differences in the mechanical properties of said components become significantly smaller (due to the similar values for the mechanical properties scaled to the density of the materials, Table 4), and the recommended manufacturing technology should be chosen considering other aspects such as component/mold complexity, setup time (since the recommended curing time is identical) and resin costs (in general, the infusion resin is more expensive than the layup resin due to the lower viscosity requirements).

The micromechanical modeling of the two types of materials using both analytical and numerical methods estimated the Young’s modulus of the fibers to be in the range of 18.5 GPa and 20.2 GPa, being in accordance with the literature data. These analyses also evaluated the air content present in the materials, correlating with the optical investigations of the outer surfaces of the materials.

For the macromechanical evaluation, the use of anisotropic plasticity according to the Hill formulation yielded relatively accurate results, especially when considering the amount of approximation used, with the resin infusion results being superior from an accuracy standpoint when compared to the wet layup results.

Regarding the numerical analyses, this study shows that accurate material models can be developed without extensive testing, reducing the time and cost expenditures associated with the process. Though the simulations provided acceptable results when compared with the available experimental data, the validation of the material models would, however, require additional tests (such as flexural loadings) and different ply stacks (i.e., [0°/45°]_n_). In addition, the mechanical testing of the finished parts should be comprehensive in order to validate the designs, due to the relatively large scatter in the experimental results and the required approximations used in the definition of the material models.

## Figures and Tables

**Table 1:** Mechanical properties of some synthetic and natural fibers [3,4,7,29,30].

Fiber	Young’s Modulus [GPa]	Tensile Strength [MPa]	Density [g·cm^−3^]
Carbon	200–500	3000–7000	1.8
Aramid	60–80	2500–3200	1.5
E-Glass	70–90	1800–4500	2.5
Flax	27–85	345–2000	1.54
Hemp	17–70	368–800	1.47
Jute	10–55	393–773	1.44

**Table 2:** Measured dimensions and evaluated densities.

Manufacturing Process	Average Thickness [mm]	Standard Deviation [mm]	Average Surface Area [mm^2^]	Standard Deviation [mm^2^]	Average Mass [g]	Standard Deviation [g]	Average Density [g·cm^−3^]	Standard Deviation [g·cm^−3^]
Wet layup	2.296	0.0921	5088.67	287.151	11.787	0.979	1.008	0.0286
Infusion	2.147	0.0615	5057.69	296.178	13.131	1.121	1.208	0.0358

**Table 3:** Average mechanical properties.

	Young’s Modulus [MPa]	St. Deviation [MPa]	Tensile Strength [MPa]	St. Deviation [MPa]
WL 0°	7850.76	637.53	53.64	2.74
WL 45°	4789.26	474.14	37.86	2.21
WL 90°	7268.75	418.144	53.07	3.35
Inf 0°	9159.99	1256.38	72.04	4.31
Inf 45°	6429.58	390.62	53.63	2.24
Inf 90°	8497.91	337.49	68.97	2.81

**Table 4:** Normalized average mechanical properties.

	E·ρ^−1^ [MPa·cm^3^·g^−1^]	σ_max_·ρ^−1^ [MPa·cm^3^·g^−1^]
WL 0°	7763.05	53.06
WL 45°	4693.37	37.09
WL 90°	7383.47	53.94
Inf 0°	7669.55	60.49
Inf 45°	5324.88	44.41
Inf 90°	6995.58	56.76

**Table 5:** Average mechanical properties of the resins.

	Young’s Modulus [MPa]	St. Deviation [MPa]	Tensile Strength [MPa]	St. Deviation [MPa]
Epoxy BK	3230.96	265.81	23.24	8.31
IN2	3013.98	342.76	48.69	2.93

**Table 6:** Intervals of variation for the anisotropic properties of the materials.

Material	$G_{12}$ $\left[GPa\right]$	$G_{13}$ $\left[GPa\right]$	$G_{23}$ $\left[GPa\right]$	$\nu_{12}$ $\left[-\right]$	$\nu_{13}$ $\left[-\right]$	$\nu_{23}$ $\left[-\right]$
Wet layup	2.37–2.45	1.86–1.88	1.82–1.84	0.245–0.249	0.311–0.312	0.321–0.322
Resin infusion	2.64–2.69	2.13–2.17	2.11–2.13	0.25–0.253	0.311–0.313	0.319–0.321

**Table 7:** Elastic properties of the two material models used in the simulations.

Material	$E_{1}$ $\left[MPa\right]$	$E_{2}$ $\left[MPa\right]$	${\nu_{12}}^{*}$ $\left[-\right]$	${G_{12}}^{*}$ $\left[MPa\right]$	${G_{13}}^{*}$ $\left[MPa\right]$	${G_{23}}^{*}$ $\left[MPa\right]$
Wet layup	7850	6800	0.247	2410	1870	1830
Resin infusion	9130	8550	0.251	2670	2180	2120

* Approximate average values from the intervals presented in Table 4.

**Table 8:** Values for the R coefficients of the two materials.

Material	$R_{11}$	$R_{22}$	${R_{33}}^{*}$	$R_{12}$	${R_{13}}^{*}$	${R_{23}}^{*}$
Wet layup	1	0.98	1	0.69	0.69	0.69
Resin infusion	1	0.93	1	0.77	0.77	0.77

* The values for $R_{33}$ were considered equal to $R_{11}$ and those of $R_{13}$ and $R_{23}$ equal to $R_{12}$ since they are required but irrelevant for this study.

## Data Availability

The original contributions presented in this study are included in the article. Further inquiries can be directed to the corresponding author.
